# Essential Oil Microcapsules Immobilized on Textiles and Certain Induced Effects

**DOI:** 10.3390/ma12122029

**Published:** 2019-06-25

**Authors:** Miruna S. Stan, Laura Chirila, Alina Popescu, Denisa M. Radulescu, Diana E. Radulescu, Anca Dinischiotu

**Affiliations:** 1 Department of Biochemistry and Molecular Biology, Faculty of Biology, University of Bucharest, 91-95 Splaiul Independentei, 050095 Bucharest, Romania; anca.dinischiotu@bio.unibuc.ro; 2 Research Institute of the University of Bucharest—ICUB, University of Bucharest, 91-95 Splaiul Independentei, 050095 Bucharest, Romania; 3 The National Research-Development Institute for Textiles and Leather Research, Textile Chemistry and Environment Protection Research Department, 16 Lucretiu Patrascanu Street, 030508 Bucharest, Romania; alina.popescu@certex.ro (A.P.); denisa.radulescu@certex.ro (D.M.R.); diana.radulescu@certex.ro (D.E.R.)

**Keywords:** essential oils, sage, rose, microcapsules, cosmetotextiles, dermal cells, biocompatibility

## Abstract

In order to obtain textile materials with potential utility in the development of cosmetic textiles, this study examined the deposition by padding of rose and sage microcapsules on woven textile structures, with different fiber compositions (100% cotton and 50% cotton/50% polyester). Cationization of the textile materials was performed to enhance the degree of uptake the pf the microcapsules on the fabrics’ surface. A commercially acrylate-based binder was used to fix the microcapsules to the textile substrate and to improve the durability against external factors. The finished textile materials were characterized in terms of their physical-mechanical characteristics. The distribution of microcapsules on the fabrics surface before and after five washing cycles and 1000 abrasion cycles was investigated by scanning electron microscopy. The biocompatibility in terms of cell viability, cell membrane integrity and inflammation status of the functionalized fabrics was evaluated on CCD-1070Sk normal human dermal fibroblasts. The cell morphology was evaluated by F-actin staining using fluorescence microscopy and no significant changes were noticed after the incubation in the presence of fabrics compared with control. The in vitro biocompatibility evaluation on human skin cells confirmed the absence of cytotoxicity after the short-term exposure, supporting further in vivo use of these innovative textiles with improved properties.

## 1. Introduction

The textile industry has recently implemented novel technologies for applications in diverse fields, mainly the cosmetic domain. Nowadays several commercial cosmetotextile products are available on the market. For cosmetic purposes, these products were designed to ensure transfer of active compounds to the skin [1]. Cosmetotextiles are fabrics embedded with active compounds as cosmetic ingredients, which are transferred and have the ability to ensure an optimum exchange in order to generate benefic effects regarding skin appearance. Through the use of cosmetotextiles, well-being and health can be improved where certain substances or even vitamins could increase skin health [2]. Moreover, these textile materials establish the basis for the delivery system of biologically active substances in contact with the skin. Active compounds (ACs) are frequently incorporated into these vehicles. Due to friction with the skin the delivery systems are deteriorated, allowing a direct release of the compounds and absorption by the skin. People with sensitive skin can benefit from these products due to the slow release of the active compounds on the skin [3].

Recently, textile applications involving the use of microencapsulation of active substances have increased. This technology enhances the durability of the encapsulated material providing some expected benefits to the textile material [4]. Therefore, the active compounds’ functionality is transferred to the material by the finishing treatment. For that reason, it is necessary to determine proper methods which will allow fixing microcapsules on the fabric for long periods of time [5].

In order to develop textile-based systems for controlled release of an active compound and achieve an efficient embedding of microcapsules into a textile substrate, there are numerous conditions that must be fulfilled to determine a suitable protection of encapsulated agents, with specific availability and controlled release rate [6].

Various active agents present in cosmetic applications are in unstable forms. They can undergo oxidation, volatilization or react with other formulation components causing skin irritation. Microencapsulation is a feasible alternative to increase the stability of these compounds [7]. The core material of the microcapsules is safely entrapped inside the shell and is released by friction, a change of temperature or pH, pressure, diffusion, biodegradation through the shell, or shell dissolution [8]. Furthermore, active compounds are advantageous in different industries, such as perfume and flavor industries, being extensively researched for their uses in pharmaceutical, cosmetic, and textile industries. Essential oils (EOs) are very sensitive to the environment and degrade easily upon exposure to oxygen, heat and light. They are hydrophobic and insoluble in water, reducing their bioavailability and absorption into the skin. To overcome these issues, adequate techniques or procedures have been developed, creating a suitable formulation of EOs to protect their properties and activities, such as anti-inflammatory, antibacterial, antifungal, analgesic, sedative, spasmolytic, antioxidant and flavoring properties [9,10].

Some essential oils that have attracted great interest and have been widely used in aromatherapy or cosmetotextiles are sage (*Salvia officinalis*) and rose (*Rosa damascena*) oils due to their main compounds. For example, the dominant compound of sage essential oil is 1,8-cineole, a substance with antifungal and antimicrobial activities [11], while in rose essential oil beneficial compounds such as citronellol, linalool, geraniol, flavonoids, and citral are found [12]. Rose essential oil (REO) presents the highest applicability and effectiveness among a wide range of plants used worldwide in traditional medicine [13]. It is understood that REOs exhibit a relaxing effect, thus leading to their usage for depression, anxiety, and stress-related conditions [14]. The pharmacological properties of REOs are numerous, including hypnotic, analgesic, antimicrobial, anti-inflammatory, anti-oxidant, emollient and moisturizing effects. Due to their antibacterial properties, REOs achieve high efficiency in moisturizing dry skin, being often recommended for facial treatments, and also for cleansing the skin from bacteria that cause acne and then hydrating the skin [15,16]. Sage essential oil (SEO) is obtained from one of the widest spread members of the *Lamiaceae* family and has been utilized as treatment against perspiration and fever, and also as carminative, spasmolytic, and wound healing agent [17]. Other studies have shown numerous other pharmacological activities of SEO, including anti-inflammatory, anti-nociceptive, antioxidant, antimicrobial and hypoglycemic effects [18,19,20]. 

Microcapsules are commonly synthesized by using degradable polymers as shell materials for drug delivery systems. The active agents are encapsulated inside the polymeric shell, allowing their controlled release over time. Particular characteristics such as the physical-chemical properties of the polymers, biodegradation kinetics, thermodynamic compatibility between polymers and ACs and the type of ACs, generally control the drug release rates [21]. A polymeric material frequently used for microencapsulation of essential oils is melamine formaldehyde (MF). MF is a kind of resin, a thermosetting polymer material, which can form different crosslinking network structures, because of its superior mechanical strength and thermal stability [22]. MF is an extremely important thermosettable material with outstanding transparency and resistance to abrasion, heat, weathering acid and alkali. Due to these advantages, melamine-formaldehyde microcapsules are used to encapsulate rose and sage essential oils [23].

To achieve a prolonged washing durability, the microcapsules must be fixed onto the textile substrates. The adhesion methods involve the use of two main groups of binders: polymeric resins, with film-forming ability, and polyfunctional cross-linking agents [24]. Often used binder types are cross-linkable silicones, polyacrylates and polyurethanes. In order to effectively bind the microcapsules to the fibers and to minimize their loss during washing, a certain amount of binder is required. Moreover, the release rate of the encapsulated cosmetic ingredients themselves can also be tailored by varying the amounts and types of binder used [25]. 

The demand for a productive and healthier lifestyle will always generate a specific market for textiles with the ability to promote the ’well-being’ of consumers. Considering future perspectives, it is desirable for cosmetotextile applications to achieve prolonged effects regarding textile functionality. The functionality of the textile products also requires a physical-mechanical and antibacterial assessment which were performed in numerous studies [26,27,28,29,30,31,32]. Many textiles are excellent media for transferring cosmetic compounds, as the essential oils-based products have a relatively lower incidence of adverse effects compared to modern synthetic compounds. For a thorough evaluation of biological properties, further analyses must be accomplished from the biocompatibility point of view. Current research studies have mainly focused on the antibacterial activity of textiles, being less concerned about the biocompatibility of the textile materials in contact with the human tissues.

The main goal of this study was to develop textile materials with cosmetic purposes by applying rose and sage melamine microcapsules on woven fabrics with different fiber compositions (100% cotton and 50% cotton/50% polyester) and to examine the influence of the overall finishing processes on the physical-mechanical characteristics, while adding as a novelty the biological properties assesment.

## 2. Materials and Methods

### 2.1. Materials and Reagents Used

100% cotton fabric with the mass per unit area of 168 g/m^2^ (Code 1) and 50% cotton/50% polyester fabric with the mass per unit area of 196 g/m^2^ (Code 2) were used for the functionalization treatments. Sage and rose aqueous-based melamine microcapsules dispersions containing 50% active compound supplied by AITEX (Alcoy Spain), with particle sizes ranging from 5 to 10 µm, have been used in order to develop textiles for cosmetic purposes. Kemapon PC/LF (Kem Color S.p.a, Settimo Torinese, Italy)—nonionic wetting agent and detergent based on fatty alcohol polyglycol ether, Kemaxil Liq. (Kem Color S.p.a.)—H_2_O_2_ stabilizer with dispersing and sequestering properties based on disodium salt of aminopolycarboxilic acid, Kemapol SR 40 Liq. (Kem Color S.p.a.)–sequestering agent for calcium and magnesium salts, dispersing and colloid protector agent have been used for preliminary treatments. Itobinder AG (LJ Specialities, Derbyshire, UK) has been used for binding of microcapsules on the surface of textile materials. Itofix EZF (polyethylene polyamine resin) from LJ Specialities was used as cationization agent for the cotton fibers. 

### 2.2. Preliminary Treatments

To ensure a proper hydrophilicity of the textile fabrics, and to ensure a high efficiency of the functionalization process, two successive preliminary preparation steps were applied: a hot alkaline treatment followed by bleaching. Hot alkaline treatment was performed at 1:10 MLR (material to liquor ratio) at 98 °C for 90 min, the treatment bath containing 8 mL L^−1^ NaOH 38°Bé, 3 g L^−1^ Na_2_CO_3_, 3 g L^−1^ Na_3_PO_4_, 2 g L^−1^ Kemapon PC/LF and 1.5 mL Kemapol SR 40 Liq. The woven fabrics were then repeatedly rinsed at 80 °C, 60 °C, 40 °C and at room temperature for 10 min. Bleaching of woven fabrics was performed at 98 °C for 60 min in the presence of 20 mL L−1 H_2_O_2_, 4 mL L^−1^ 38°Bé NaOH, 2 mL L^−1^ L, H_2_O_2_ stabilizer, Kemaxil Liq., 1 mL L^−1^ Kemapon PC/LF. Cationization of textile materials was performed in order to modify the surface electric charge of cotton fibers, introducing positively charged sites (cationic groups), aiming to enhance the uptake degree of the microcapsules on the fabrics surface. Textile materials were padded with 10g L^−1^ Itofix EZF and then dried at 100 °C for 2 min.

### 2.3. Treatments Functionalization

Microcapsules in the forms of aqueous dispersion were applied on textile materials at a laboratory scale by using a laboratory scale padder (ROACHES, West Yorkshire, UK) reproducing the industrial scale application conditions. The fabrics were padded in concomitant phase with 30 g L^−1^ microcapsules dispersions and 80 g L^−1^ Itobinder AG, at 80% wet pick-up, dried at 100 °C for 2 min followed by curing at 150 °C for 2 min. Drying and curing operations have been performed on the drying/curing/heat-setting unit, model TFO/S 500 mm (ROACHES). The sample codification system is presented in Table 1.

### 2.4. Characterization Methods

#### 2.4.1. Mechanical Tests

The treated fabrics were characterized in terms of the main physical-chemical and physical-mechanical characteristics, respectively: mass per unit area (SR EN 12127-2003), number of thread per unit length (SR EN 1049-2: 2000-Method A, B), maximum force (SR EN ISO 13934-1/2013), elongation at maximum force (SR EN ISO 13934-1/2013), water vapor permeability (STAS 9005: 1979), permeability to air (SR EN ISO 9237: 1999).

#### 2.4.2. Treatments Durability

##### Washing Durability

For the testing of treatments washing durability the treated samples were cut in 5 cm × 5 cm pieces and then have been subjected to five washing cycles under the following conditions: 2 g·L^−1^ detergent containing no phosphate and bleaching agent, at a temperature of 40 °C for 30 min. Samples were subsequently rinsed with cold deionized water and freely dried horizontally. The washed samples were then analyzed by scanning electron microscopy (SEM, FEI Europe B.V., Quanta 200, Eindhoven, The Netherlands). 

##### Abrasion Durability 

According to the SR EN ISO 12947-2/2017 standard, the treated fabrics were tested by a Martindale Abrasion Tester (James Heal, West Yorkshire, UK) for 1000 abrasion cycles. The presence of the microcapsules after an abrasion test was observed by SEM analysis. 

#### 2.4.3. Scanning Electron Microscopy (SEM)

SEM analysis was used to investigate the distribution of microcapsules on the fabrics surface after 5 washing cycles and 1000 abrasion cycles, respectively. A Quanta 200 Scanning Electron Microscope (FEI Europe B.V., Quanta 200, Eindhoven, The Netherlands) equipped with a GSED detector and an accelerating voltage of 12.5 kV–20 kV has been used in this respect.

### 2.5. In Vitro Dermal Cell Response to Fabrics Exposure

#### 2.5.1. Cell Culture and Exposure to Fabrics

CCD-1070Sk cells (CRL-2091, ATCC, Manassas, VA, USA), which are dermal fibroblasts harvested from normal dermal tissue from a male newborn, were used in the in vitro assays performed for this study. These were maintained at 37 °C in a humidified atmosphere with 5% CO_2_, and the growth medium used was Minimum Essential Media (MEM) with 10% fetal bovine serum. In order to assess the effect induced by textile materials to dermal fibroblasts, square cuts of 1 cm^2^ were cut from all types of fabrics. Subsequently, the materials were sterilized by exposure to ultraviolet radiation for 3 h. After seeding the cells in 24-well plates at a density of 2 × 10^4^ cells/cm^2^, they were allowed to adhere for 24 h, and then the sterilized fabrics were added using a tweezer. In parallel, fibroblasts were cultivated also at the same density on cell culture inserts for 24-well plate with 3 µm pore size and polyester track-etched membrane (Corning Inc., Corning, NY, USA). After one week of cell growing, the fabrics were added in the basal compartment. All plates were incubated with the samples for 12 h in the interval of 8 PM–8 AM to simulate a nocturnal exposure similar to the wearing of textiles such as pajamas. After this time, the medium was harvested and the cells were washed with PBS and processed appropriately for the biocompatibility tests [33].

#### 2.5.2. Cell Viability Assay

The principle of the MTT [3-(4,5-dimethylthiazol-2-yl)-2,5-diphenyltetrazolium] assay is based on the dehydrogenase activity of live cells of reducing the MTT yellow colored compound to a dark blue formazan. The reduction factor is directly proportional to the number of viable cells, representing an index of cellular integrity. After removing the medium, the cells were washed with PBS, MTT solution (1 mg/mL) was added, and incubated at 37 °C for 2 h in the dark. The MTT solution was removed, and the formazan crystals were resuspended in isopropanol. The measurement of absorbance was performed at a wavelength of 595 nm. MTT assay was used only to quantify the viability in 24-well cell culture plates as it is indicated in the literature. Therefore, we have used the actin and nuclei staining to show a qualitative expression of viability for cultures on inserts.

#### 2.5.3. Nitric Oxide (NO) Level Measurement

The concentration of NO in the culture medium was determined using the Griess reagent. A volume of 80 μL of the culture media collected after the exposure was pipetted into 96-well plates. A stoichiometric solution (*v*/*v*) of 1% sulphanilamide (Sigma-Aldrich, St. Louis, MO, USA) and 0.1% dihydrochloride N-(1-naphthyl) ethylenediamine (Sigma-Aldrich) was prepared just before and it was added to wells in a ratio of 1:1 (*v*/*v*). The absorbance of the colored product formed was measured spectrophotometrically at 550 nm at the Flex Station plate reader (Molecular Devices, San Jose, CA, USA), and the NO concentration in the samples was determined by extrapolation on a standard sodium nitrite (Sigma-Aldrich) curve with concentrations between 3125 and 100 μM, which was carried out under the same conditions.

#### 2.5.4. Lactate Dehydrogenase (LDH) Release Assay

This LDH cytotoxicity assay is based on the reduction of NAD^+^ to NADH, as the cell membrane destruction leads to the LDH release into the culture medium. This can be quantified by coupled reactions in which the enzyme catalyzes the conversion of lactate into pyruvate by reducing NAD^+^ to NADH, being subsequently used to reduce a tetrazolium salt—2-[4-iodophenyl]-3-[4-nitrophenyl)-5-phenyltetrazolium chloride (INT)—to a red formazan which can be measured spectrophotometrically at the wavelength of 490 nm. Determination of LDH release was performed with the Cytotoxicity Detection Kit^PLUS^ (Roche, Mannheim, Germany) according to manufacturer’s instructions. A volume of 50 μL of culture medium harvested after 12 h of exposure to textiles was incubated with 100 μL of a reaction mixture containing a catalyst and a colorant for 20 min at room temperature in the dark. Absorbance was read at a Flex Station multireader at 490 nm.

#### 2.5.5. F-actin Staining

At the end of incubation, the fibroblasts were fixed with 4% paraformaldehyde for 20 min and permeabilized with 0.1% Triton X-100–2% bovine serum albumin for 1 h. The actin filaments were stained with 10 µg/mL phalloidin-fluorescein isothiocyanate (FITC) and the nuclei were counterstained with 2 µg/mL 4′,6-diamino-2-phenylindole (DAPI). The cells were visualized with an IX71 inverted fluorescence microscope (Olympus, Tokyo, Japan).

### 2.6. Statistical Evaluation of In Vitro Data

All in vitro results were statistically analyzed using one-way ANOVA followed by Bonferroni’s post-hoc test (GraphPad Prism 5, GraphPad, San Diego, CA, USA) and expressed as mean ± standard deviation (SD) (n = 3). A value of *p* less than 0.05 was considered statistically significant.

## 3. Results and Discussion

### 3.1. Physical-Mechanical Characteristics

The main physical-mechanical and comfort characteristics of 50% cotton/50% polyester and 100% cotton fabrics treated in various experimental variants with dispersions of microcapsules containing rose/sage essential oil are shown in Table 2.

In terms of physical and mechanical characteristics, the obtained results revealed that the applied functionalization treatment led to the fabrics’ shrinkage, for both fabric variants leading to increased values of the mass and density, which is a normal behavior for textile materials treated in wet liquor and with high curing temperatures.

A decreasing of maximum force was recorded in the warp direction only, with about 5% lower values in the case of 100% cationized cotton fabric and of about 10% in the case of cationized cotton/polyester blended fabric, showing a minimum influence of the functionalization treatment on fabric strength. The cationization process influenced much more the maximum force without a significant reduction of this characteristic. 

Air and water vapor permeability recorded lower values compared to the untreated fabric, due to the polymeric substances deposited at the surface of the fabric in the form of a semi-permeable film, indicating a comfort decrease for all selected experimental variants. The immobilized microcapsules would fill the gap between yarns letting the airflow pass less easily through the treated fabrics.

### 3.2. SEM Analysis

In order to evaluate the degree of microcapsules immobilization on the textile materials surface, provided by the cationization pre-treatments or by the binder added in the treatment baths, the treatments washing durability has been assessed through SEM analysis.

**Figure 1:**
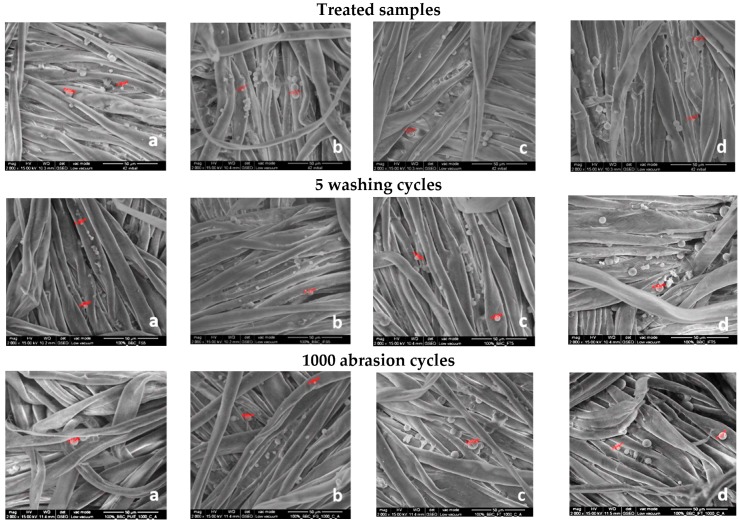
SEM images recorded at 2000× magnification for 100% cotton fabrics treated in different experimental variants with sage (S) and rose (R) microcapsules: (**a**) 1V_1_S, (**b**) 1V_2_S, (**c**) 1V_1_R, (**d**) 1V_2_R.

**Figure 2:**
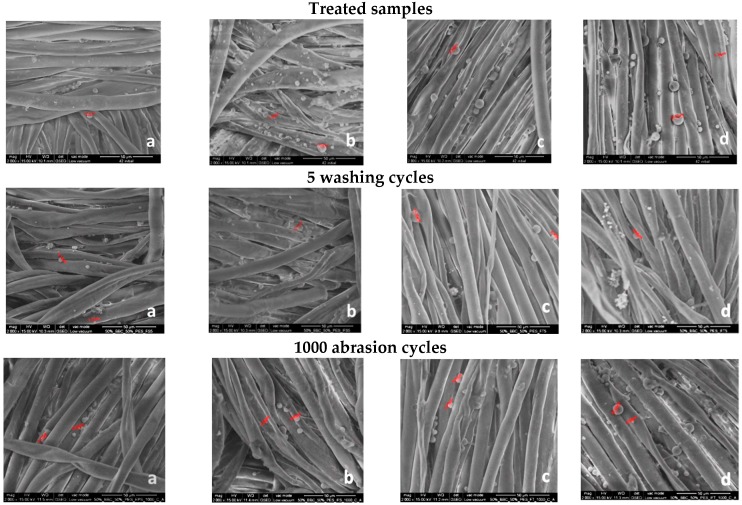
SEM images recorded at 2000× magnification for 50% cotton/50% polyester fabrics treated in different experimental variants with sage (S) and rose (R) microcapsules: (**a**) 2V_1_S, (**b**) 2V_2_S, (**c**) 2V_1_R, (**d**) 2V_2_R.

Figure 1 and Figure 2 present the SEM images recorded at 2000× magnification on textile fabrics before and after five washing cycles and after 1000 abrasion cycles, respectively. The SEM images recorded for treated fabrics, in different experimental variants, highlight the fact that microcapsules have been successfully immobilized on the textile fabrics surface. A higher numbers of microcapsules has been observed for the samples subjected to the cationization operation, proving the usefulness of this operation. Some microcapsules are deformed, collapsed or broken, or are in the form of conglomerates of various sizes, adhering to the fabrics surface. Electronic images recorded after five washing cycles reveal the presence of a lower number of microcapsules adhered to the fabrics’ surface compared to unwashed samples. During washing tests, some of the microcapsules were removed from the fibers, some of them were broken, leaving only wall material residues still adhered to the fiber’s surfaces. Also, it could be observed that binder fixation was successfully achieved because the residues of microcapsules still adhered to the fibers after five washing cycles. 

The abrasion test acted as a simulation of friction between the functionalized samples and human body. The number of microcapsules distributed on the surface is lower after 1000 abrasion cycles, only few microcapsules were still found adhering to the fibers surface as shown in Figure 1 and Figure 2. It was found that some microcapsules were broken leaving the residues of microcapsules still adhered to the fiber surface. 

### 3.3. Biocompatibility Evaluation

Before their placement on the market, textile materials impregnated with essential oil microcapsules and having improved antioxidant and rejuvenating properties, should be carefully evaluated to check if they are biocompatible and safe for the skin following direct contact [34]. The cosmetotextile industry is rapidly growing and it is very important to provide non-toxic products for daily human use.

In order to assess the biocompatibility of these treated textiles, we have used two types of cell culture conditions, namely 24-well culture plates with inserts or without them. The use of trans-well inserts allows the possibility to put the textiles in the basal compartment below the membrane and the cells will not stick on the textiles. By contrast, the addition of treated textiles on the top of the cells can create high pressure on fibroblasts, giving unreliable results. However, we have tested both approaches from the point of view of cell viability and cytotoxicity as it is regulated for in vitro screening [35] in order to compare the results between them and with cells cultured without any fabric (“control cells”).

The types of assays used in our experiments are part of the battery of cytotoxicity tests described in ISO 10993-5:2009, part 5 [36,37]. Taking into account the diversity of medical devices and the complexity of the interaction between the medical devices and body, a uniform evaluation method or cytotoxicity test evaluation system cannot be established [38]. The cytotoxicity methods selected by us, such as the detection of cell damage by morphological changes (by F-actin staining), determination of cell damage (by lactate dehydrogenase release measurement), measuring cell growth and metabolic properties (by MTT or WST assay), represent the most commonly used tests for in vitro biocompatibility investigation of medical devices, including functionalized textiles, according to the literature available at this moment [39,40,41,42]. It is well known that there are three types of cytotoxicity tests mentioned in ISO 10993-5: extract, direct contact and indirect contact tests, the second one being the most sensitive for testing the cytotoxicity of the medical devices [38]. In this way, we have selected the direct contact evaluation with the traditionally cell cultures of planar, static 2D models and the new 3D model on trans-well culture system in order to bring new evidences for helping this area of research. Most probably, the next step in improving the methodology of textiles’ cytotoxicity assessment will be the use of 3D bioprinted skin models, providing a more uniform model which better mimic the in vivo skin and with lower costs of production compared to currently available models on the market, such as Epiderm™, SkinEthic RHE™, and EpiSkin™ [43].

We decided to select a 12 hour-exposure time in order to mimic one long sleep in pajamas, as the average sleep lasts between 7 and 9 h. A longer interval would not be relevant as results may be affected by other skin reactions (such as textile dermatitis, infections) and dermatologists do not recommend wearing the same clothes for more than half a day and to change them the next day [44]. Furthermore, pajamas are strongly associated with the sleep time, and help us to mentally prepare for bedtime; so, wearing them during the day can alter people’s productivity. According to Hygiene for Health, specialists recommend not to prolong this reasonable period of 12 h because clothes can harbor microorganisms, spread bad odors, and affect our psychical (mental) disposition and social interactions. 

**Figure 3:**
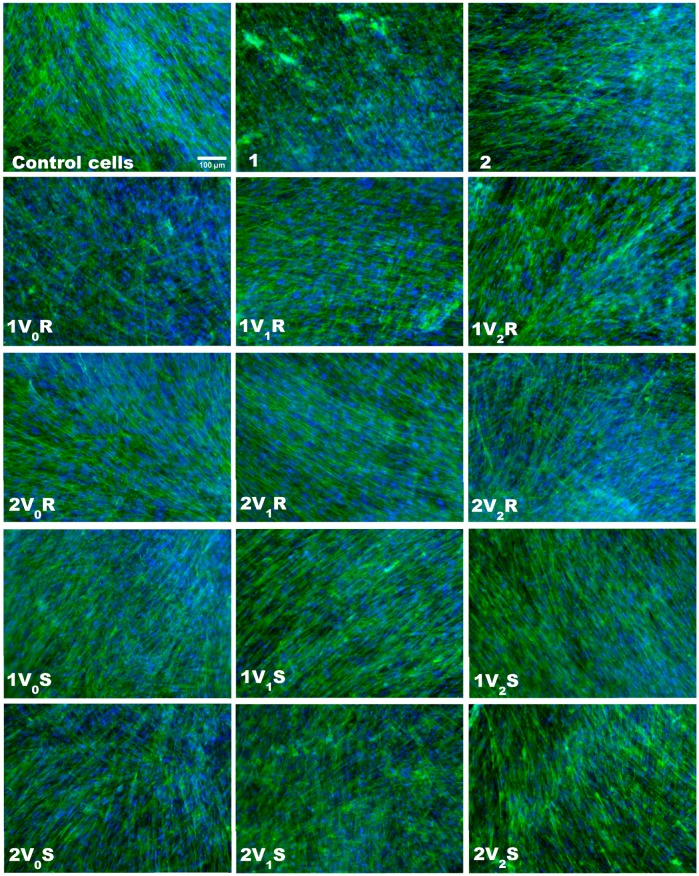
Fluorescence images of actin filaments (green) and nuclei (blue) staining in human dermal fibroblasts cultured on inserts in the presence of fabrics treated in different experimental variants with sage (S) and rose (R) microcapsules for 12 h. Scale bar of 100 µm shown on first micrography is the same for all images.

**Figure 4:**
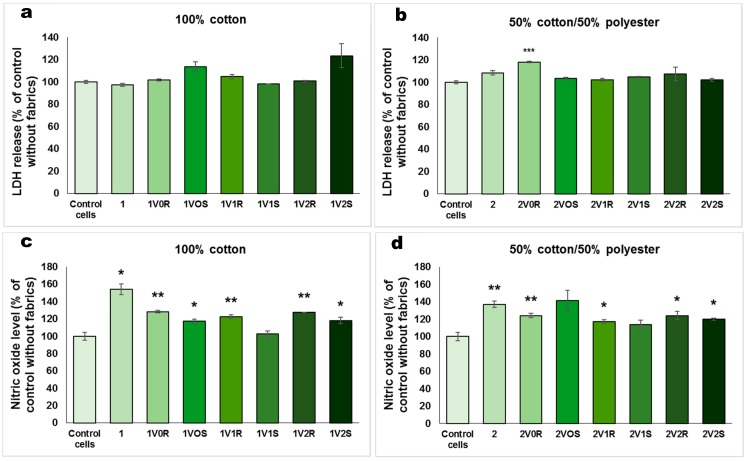
Biocompatibility in terms of LDH release (**a**,**b**) and NO level (**c**,**d**) for 100% cotton and 50% cotton/50% polyester fabrics treated in different experimental variants with sage (S) and rose (R) microcapsules, that were incubated for 12 h with human dermal fibroblasts cultured on inserts. Data are represented relative to control cells without fabrics as mean ± standard deviation (SD) (n = 3). * *p* < 0.05, ** *p* < 0.01 and *** *p* < 0.001 compared to the control. In order to evaluate the biocompatibility of treated fabrics in a classic cell culture plate without inserts, the cell viability (Figure 5a,b), LDH release (Figure 5c,d) and NO level (Figure 5e,f) were assessed. In contrast with the good cell viability observed for the experiment performed in cell culture inserts (Figure 3), the results obtained after 12 h of exposure with textiles on the top of the cells showed a decrease in cell number compared to control. The cell viability measured for the materials functionalized by cationizing and padding was by 40–50% lower than for the control. Taking into account that the viability was even lower for the untreated 100% cotton fabric, it could be suggested that the functionalized textiles better protected the fibroblasts attachment on the surface of culture plate.

The viability and morphology of skin cells cultured on inserts in the presence of treated fabrics was evaluated by staining the actin filaments and nuclei (Figure 3). The fluorescence images showed a very good viability for all types of treated fabrics, being comparable with that of control cells and untreated fabrics. Cell spreading was not affected after 12 h of exposure to these fabrics, and the actin filaments presented a normal morphology and alignment in the fibroblasts. The cell membrane integrity was checked by LDH test and the results were shown in Figure 4a,b. The values obtained for cells incubated with treated fabrics were almost similar with those measured for control cells. However, the highest LDH release was measured for 1V_2_S and 2V_0_R, the level being increased with 23% and 18%, respectively, compared to control cells. These results confirmed that essential oils microcapsules immobilized on textiles did not induce LDH release as the cell membranes was not injured during the incubation of 12 h with these types of modified fabrics. 

Inflammatory potential of the fabrics was analyzed by measuring the level of NO in culture media (Figure 4c,d). The NO production normalized to cell density for the control sample was 2.72 ± 0.28 nmol/10^4^ cells. Although the highest levels of NO released in the culture medium (154% and 134% compared to control cells) were obtained for untreated fabrics (100% cotton and 50% cotton/50% polyester, respectively), the samples treated by cationization and padding did not induce a release of NO with more than 27% of the value measured for control cells. As this molecule is an indicator of inflammation [45], we can suppose that these types of treated fabrics did not trigger inflammatory reactions in dermal fibroblasts during 12 h of exposure.

**Figure 5:**
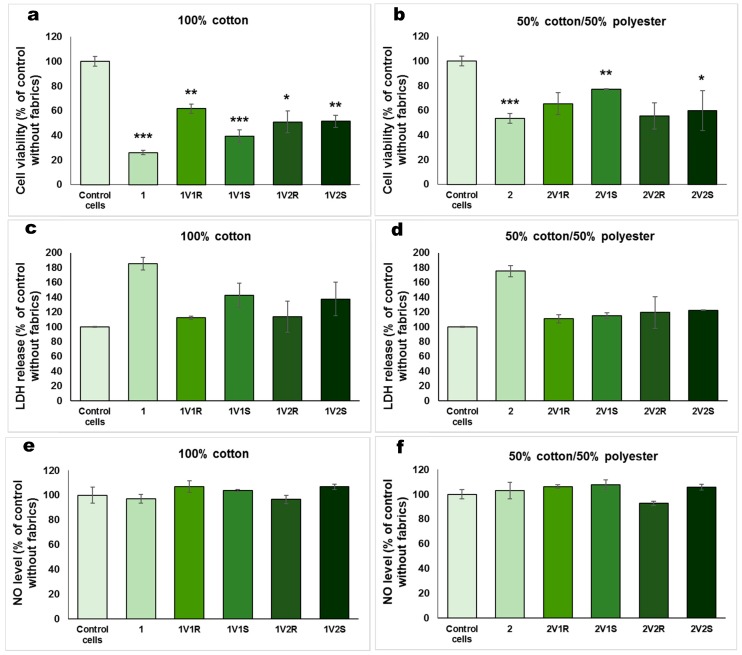
Biocompatibility in terms of cell viability (**a**,**b**), LDH release (**c**,**d**) and NO level (**e**,**f**) for 100% cotton and 50% cotton/50% polyester fabrics treated in different experimental variants with sage (S) and rose (R) microcapsules, that were incubated for 12 h with human dermal fibroblasts cultured without inserts. * *p* < 0.05, ** *p* < 0.01 and * *p* < 0.001 compared to the control.

The incubation with textiles in 24-well culture plates without inserts can have several disadvantages that can affect the cell viability. The cells can stick on the textiles during the exposure. The textiles sitting on the top of the cells can apply a high pressure on fibroblasts affecting their normal spreading, and the manipulation of textile in the well can facilitate the detachment of several cells, explaining the decrease in cell viability shown in Figure 5a,b.

In the case of the effect induced on the release of LDH from the cells, it was revealed that the 50% cotton/50% polyester fabrics treated with essential oil microcapsules did not significantly alter the integrity of the cell membrane, the obtained values being close to the control without fabrics. Slight increases were observed for 100% cotton fabrics treated with sage essential oil microcapsules (a 42% and 37% increase compared to control for 1V_1_S and 1V_2_S, respectively). It is very important to highlight that the untreated fabrics induced a significant LDH release from cells, suggesting the alteration of cell membranes during the 12 h exposure. These findings correlate very well with the cell viability decreased observed for these blank fabrics.

Regarding the level of NO released in the extracellular environment (Figure 5e,f), there were no significant changes compared to control, confirming the absence of inflammatory potential of these functionalized fabrics. The NO production normalized to cell density for control sample was 1.83 ± 0.48 nmol/10^4^ cells.

Regarding the research on functionalized textiles, most of the studies were focused on the immobilization of nanoparticles or dyes onto fabrics. Biocompatibility has been reported for several textiles impregnated with different nanoparticles (copper oxide, silver or titanium dioxide) [33,41,46] which were designed for industrial and commercial applications due to their bactericidal activity. Also, the textile technologies have succeeded to bring novel advantages as researchers were able to develop biocompatible core-shell composite living fibers to act as scaffold for tissue engineering purposes [39]. Furthermore, fabrics without cytotoxic effects were developed for medical purposes, such as nonwoven polymeric fibrous bandaging materials for the treatment of infected wounds [40] and polypyrrole-coated polyester fabrics for vascular prostheses [42].

## 4. Conclusions

The melamine-based microcapsules with sage or rose essential oil content were embedded onto the cotton and cotton/polyester blended fabrics by using the pad-dry-cure method. The minimal influence of the treatments on the main physical and mechanical characteristics of woven fabrics was confirmed. The comfort indicators (air and water vapor permeability) recorded lower values compared to the untreated fabric, which it was to be expected when depositing polymeric substances on the textile surfaces. A higher reduction of this characteristic was obtained for the pre-cationized samples. However, the values for permeability to air and water vapor permeability, for all analyzed samples, fulfill the minimum requirements for skin contact clothing articles and home clothing (min. 30 L/m^2^/s for permeability to air and min 25% for water vapor permeability). The functionalization treatments with rose/sage microcapsules withstood five washing cycles, the washing durability being acceptable. Based on the biocompatibility tests, it was revealed that in the presence of functionalized fabrics the fibroblasts cultured on inserts maintained their viability, membrane integrity and NO concentration at basal levels. As our findings showed their good biocompatibility, these newly obtained textiles with essential oils microcapsules immobilized on them can become an ideal candidate for the cosmetotextile industry, providing certain biological properties, such as antioxidant, anti-inflammatory, antibacterial and flavoring effects. 

## Figures and Tables

**Table 1:** Sample codification.

Code	Microcapsules	Textile Fabrics	Cationization	Binder	Padding
1	-	100% cotton	−	−	−
2	-	50% cotton/50% polyester	−	−	−
1V_0_S	Sage	100% cotton	−	−	+
1V_1_S	Sage	100% cotton		+	+
1V_2_S	Sage	100% cotton	+	+	+
2V_0_S	Sage	50% cotton/50% polyester	−	-	+
2V_1_S	Sage	50% cotton/50% polyester	−	+	+
2V_2_S	Sage	50% cotton/50% polyester	+	+	+
1V_0_R	Rose	100% cotton	−	−	+
1V_1_R	Rose	100% cotton	−	+	+
1V_2_R	Rose	100% cotton	+	+	+
2V_0_R	Rose	50% cotton/50% polyester	−	−	+
2V_1_R	Rose	50% cotton/50% polyester	−	+	+
2V_2_R	Rose	50% cotton/50% polyester	+	+	+

**Table 2:** Physical-mechanical characteristics of the treated sample.

Code	Mass (g/m^2^)	Density (Threads no/10 cm)		Maximum Force (N)		Elongation to Maximum Force (%)		Permeability to Air (l/m^2^/s)	Water Vapor Permeability (%)
		Warp	Weft	Warp	Weft	Warp	Weft		
1V_1_S	180	310	208	808	495	13.11	14.52	97.04	32.3
1V_2_S	184	312	212	733	516	13.06	14.71	78.19	30.04
2V_1_S	224	315	202	1311	719	19.24	21.3	55.8	32.9
2V_2_S	228	313	210	1245	734	22.8	20.6	54.97	31.9
1V_1_R	182	305	207	797	526	13.23	13.95	96.48	31.6
1V_2_R	185	305	214	757	520	13.70	15.21	82.38	29.9
2V_1_R	224	310	200	1284	707	19.81	18.37	63.63	31.6
2V_2_R	224	310	208	1190	704	21.7	18.95	52.88	30.7
1	168	296	210	775	475	10.57	13.85	159	44.0
2	196	304	200	1321	703	15.26	15.26	112	42.4
